# Equine obesity: beyond the equine metabolic syndrome

**DOI:** 10.1186/1751-0147-57-S1-K2

**Published:** 2015-09-25

**Authors:** Caroline Argo

**Affiliations:** 1 School of Veterinary Medicine, University of Surrey, Guildford, Surrey, UK

## 

Laminitis is a common and debilitating condition which initially presents as an acutely painful condition of the feet and often warrants euthanasia. The condition has multiple aetiologies but acute pasture-associated laminitis is most frequently encountered and is often recurrent. The incidence and impact of laminitis led to the identification of derangements of carbohydrate and lipid metabolism and generalised or regional obesity as key risk factors for the disease. The conflation of obesity, insulin resistance and a susceptibility to laminitis is common and cases which present with these signs are considered to possess the Equine Metabolic Syndrome (EMS) phenotype. However, although the pathophysiology of laminitis, obesity and insulin regulation are linked, not all laminitic horses/ponies are obese and/or insulin resistant. Obesity has both direct and indirect negative impacts on equine health. Changes in the role of horses, away from productivity and towards the leisure/companion sector have been reflected by dramatic increases in the incidence of obesity. However, increased obesity has not been matched by an increased incidence of laminitis. Trigger factors for laminitis are likely to be multifactorial and include: season, genetic factors, diet, the gastro-intestinal and neuroendocrine regulation of metabolism and appetite, management systems, generalised and regional adiposity. Variation in the magnitude and exposure of individuals across the spectrum of risk factors likely contributes to overall risk. To date, the management of body fat content is our primary treatment modality in the prevention of laminitis and the correction of insulin resistance and obesity, whether these are encountered alone or within the EMS.

